# Cellular Features Revealed by Transverse Laser Modes in Frequency Domain

**DOI:** 10.1002/advs.202103550

**Published:** 2021-11-28

**Authors:** Zhen Qiao, Hongmei Xu, Na Zhang, Xuerui Gong, Chaoyang Gong, Guang Yang, Sing Yian Chew, Changjin Huang, Yu‐Cheng Chen

**Affiliations:** ^1^ School of Electrical and Electronic Engineering Nanyang Technological University 50 Nanyang Ave. Singapore 639798 Singapore; ^2^ School of Mechanical and Aerospace Engineering Nanyang Technological University 50 Nanyang Ave. Singapore 639798 Singapore; ^3^ School of Chemical and Biomedical Engineering Nanyang Technological University 62 Nanyang Drive Singapore 637459 Singapore; ^4^ Lee Kong Chian School of Medicine 11 Mandalay Road Singapore 308232 Singapore

**Keywords:** cell curvature, cell lasers, cell morphology, frequency spacing, hyperspectral imaging, transverse modes

## Abstract

Biological lasers which utilize Fabry–Pérot (FP) cavities have attracted tremendous interest due to their potential in amplifying subtle biological changes. Transverse laser modes generated from cells serve as distinct fingerprints of individual cells; however, most lasing signals lack the ability to provide key information about the cell due to high complexity of transverse modes. The missing key, therefore, hinders it from practical applications in biomedicine. This study reveals the key mechanism governing the frequency distributions of transverse modes in cellular lasers. Spatial information of cells including curvature can be interpreted through spectral information of transverse modes by means of hyperspectral imaging. Theoretical studies are conducted to explore the correlation between the cross‐sectional morphology of a cell and lasing frequencies of transverse modes. Experimentally, the spectral characteristics of transverse modes are investigated in live and fixed cells with different morphological features. By extracting laser modes in frequency domain, the proposed concept is applied for studying cell adhesion process and cell classification from rat cortices. This study expands a new analytical dimension of cell lasers, opening an avenue for subcellular analysis in biophotonic applications.

## Graphical abstract

This study reveals the key mechanism governing the frequency distributions of transverse modes in single cell biological lasers. Morphological information of cells can be interpreted through hyperspectral imaging of laser modes. Applications including cell classification and monitoring of cell adhesion are explored and demonstrated.

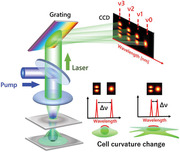

## 1 Introduction

Lasers are the pillar of modern optics, which plays an important role in amplifying weak signals in many applications.^[^ ^1^ ^]^ By integrating biological materials into the gain medium and/or micro‐cavities, the concept of biological lasers became an emerging approach in biophotonics over the past decade.^[^ ^1^, ^2^, ^3^, ^4^, ^5^, ^6^, ^7^, ^8^, ^9^, ^10^, ^11^, ^12^, ^13^, ^14^ ^]^ Due to the strong light‐matter interactions and light confinements provided by an optical cavity, subtle biological changes could be amplified during repeated interactions between the gain and cavity. The output laser, therefore, can carry important biological information, which is recorded by distinct lasing characteristics, such as narrow‐linewidth spectrum, lasing thresholds, laser modes, and emission intensities. Since the first invention of biological cell laser in 2011,^[^ ^2^ ^]^ cellular lasers have aroused great attention due to their potential applications in cell analysis, phenotyping, and detection. Thus far, various types of cavities for cellular lasers have been proposed, including whispering gallery mode (WGM)‐based cavities that are embedded into cells,^[^ ^13^, ^15^, ^16^ ^]^ and Fabry–Pérot (FP) cavities which encapsulate the cells in microcavity.^[^ ^17^, ^18^, ^19^ ^]^ The former was utilized to emit WGM‐lasing peaks for precise cell tracking and intracellular analysis. The latter, that is, FP cavity‐type, possesses the advantages of non‐invasiveness and whole‐body interactions between stimulated emission and cells, which may provide intrinsic information of the cell. In a FP cellular laser, the cell serves as a “lens” in the cavity due to higher refractive index (RI) than its surrounding medium, resulting in a distinct output laser pattern that is composed of a series of transverse modes.^[^ ^2^, ^17^ ^]^ Due to the principle of laser modes, transverse modes possess a high correlation with the physical properties of cells. Given the great progress of FP cellular lasers throughout the last decade, most lasing signals lack the ability to provide key information about the cell due to the high complexity of transverse modes. The missing key, therefore, hinders it from practical applications in biology and medicine. To bring cell laser to a next level, revealing the hidden bioinformation in cellular laser is, hence, a crucial issue.

In principle, transverse modes are eigen solutions of Helmholtz equation that governs laser oscillations in a FP cavity, where a cell provides the intra‐cavity boundary condition. Under a fixed cavity length and surrounding environment, the cell determines the attributes of transverse modes, including not only the patterns but also the lasing frequencies (or wavelengths). According to the resonance condition, the single‐pass phase shift of a laser beam in a FP cavity must be integer multiples of *π*. Such phase shift is contributed by two terms: one is the traveling‐wave phase shift (*kz*, where *k* is wave number and *z* is traveling distance); and the other is the Gouy phase shift (Δ*ϕ*_G_) which is induced by the light focusing effect of a cell.^[^ ^20^ ^]^ Traveling‐wave phase shift determines the free spectral range of longitudinal modes. To be more precise, the Gouy phase shift determines the transverse‐mode frequency spacing (frequency separations between transverse modes with adjacent orders). The fact that a cell plays the key role in focusing a transverse mode in a cell laser suggests that the frequency distribution of output transverse modes represents the physical characteristics of a cell, especially the cell morphology. However, little information has been unlocked in such principle.

In this study, we revealed the key mechanism governing the frequency distributions of transverse modes in cellular lasers. Theoretical studies were conducted to explore the correlation between the cross‐sectional morphology of a cell and lasing frequencies of transverse modes, in which the decreasing curvature of a cell shortens the transverse‐mode frequency spacing. In our experiments, cell models (water‐based droplets) and biological cells (both live and fixed adherent cells) were employed in FP cavities; meanwhile, hyperspectral images of the output laser were recorded to show how the frequency distribution of laser modes varies with cell morphology. By extracting useful frequency information, we systematically demonstrated that transverse modes can be applied for recording the spreading behaviors of C2C12 cell line and also for the classification of cortical neurons and astrocytes obtained from the rat central nervous system. This work paved a further way for cell analysis based on transverse modes from biological cell lasers that have never been revealed before.

## 2 Results

### 2.1 Optical Principle

**Figure 1:**
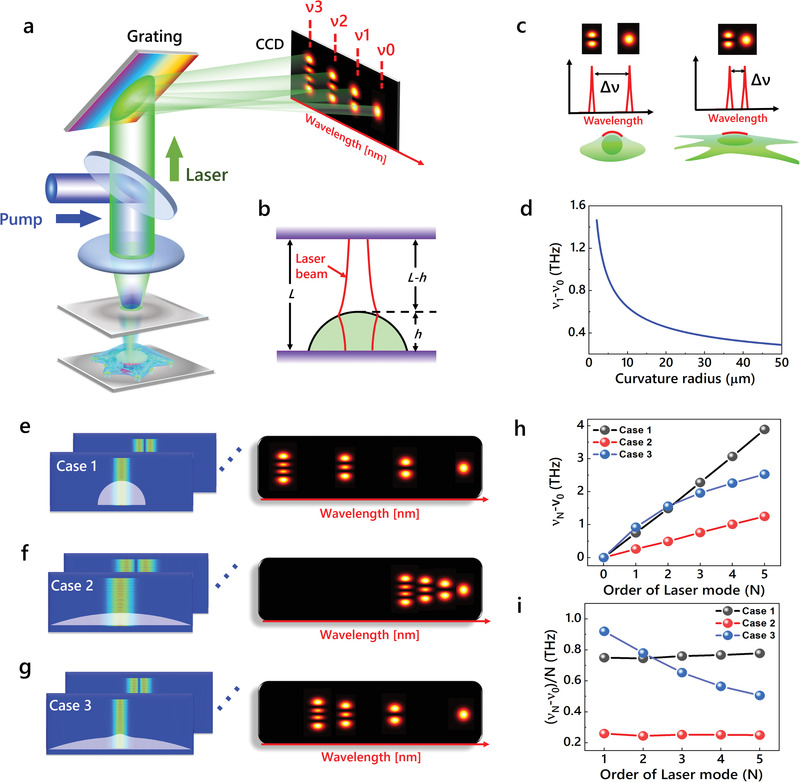
Principle of hyperspectral laser imaging of transverse modes for cell analysis. a) Schematic of a single cell laser and an imaging spectrometer. Fluorescent‐labeled cells with different morphologies are sandwiched within a FP cavity. By diffracting the output laser with the grating, a hyperspectral image of laser modes with specific frequencies (or wavelengths) will be obtained. *ν*_N_, lasing frequency of TEM_0N_ mode. b) Schematic of laser oscillation in a FP cavity. *L*, cavity length. *h*, cell height. c) Schematic illustration of frequency spacing between TEM_01_ and TEM_00_ modes (*ν*_1_ − *ν*_0_) governed by local curvature of cell. d) Calculated frequency spacing between TEM_01_ and TEM_00_ modes (*ν*_1_ − *ν*_0_) as a function of local curvature radii of cells. e‐g) Simulations on the oscillations of transverse modes in cell lasers under various cell profiles (left column) and the schematic diagrams of hyperspectral images (right column). White filled: cell profiles. e) Case 1: cell with a smooth profile and large curvature. f) Case 2: cell with smooth profile and small curvature. g) Case 3: cell with uneven profile and larger local curvature at the tip. h) Frequency distribution of laser modes (*ν*_N_ − *ν*_0_) under the cases in (e–g). i) Average frequency spacing [(*ν*_N_ − *ν*_0_)/N] under the cases in (e–g).

**Figure** **1** illustrates the principle of recording a cellular laser by transverse modes in the frequency domain. In this study, cell lasers were prepared by labeling fixed/live cells with fluorescent probes and confined in a FP cavity (Figure 1a). Details of the experimental setup can be found in Figure S1, Supporting Information. By pumping the cell at its optimal excitation wavelength, transverse modes with specific lasing wavelengths would emit from the cell laser. An imaging spectrometer was employed to obtain the frequency information of transverse modes (Figure 1a). By diffracting the output laser with a grating, a hyperspectral image of laser modes was recorded by a CCD, in which *v_N_* was defined as the frequency of *N*th‐order transverse mode (Figure 1a). The frequency information of transverse modes is highly correlated with the cell morphological features. Explicitly, the frequency spacing between TEM_01_ and TEM_00_ modes (*v*_1_ − *v*_0_) is mainly controlled by the local curvature at the top surface of a cell. In addition, the frequency distribution as a function of mode order *N* (defined by *v_N_* − *v*_0_) is governed by the whole cell. As such, we also defined the average frequency spacing of a higher‐order mode [Δ*v*_ave_ = (*v_N_* − *v*_0_)/*N*] to characterize the cell morphology in a larger area.

We first conducted the theoretical analysis to reveal how the local cell morphology governs the transverse‐mode frequency spacing. Considering that the *N*th‐order transverse mode oscillates in a FP cavity (Figure 1b), the single‐pass phase shift must be integer multiples of *π*. As such, the following relationship must be satisfied:

(1) $$\int_{0}^{L}k_{N}dz-\Delta \phi_{N,G}=q\pi$$

In Equation (1), *k_N_* is the wavenumber of *N*th‐order transverse mode equaling 2*πn*(*z*)*ν_N_*/*c*, where *n*(*z*) is the RI distribution along *z*‐direction, *v_N_* is the lasing frequency, and *c* is the light velocity; *L* is the cavity length; Δ*ϕ_N_*_,_ *_G_* is the Gouy phase shift of the laser; and *q* is a positive integer. Note that the order *N* is a unified index that equals to *m* + *n* for a Hermite Gaussian (HG*_mn_*) mode. Due to the lens effect of a cell in a cavity, the Gouy phase shift Δ*ϕ_N_*_,_ *_G_* is introduced in the processes of laser divergence and convergence (Figure 1b), which is defined by:^[^ ^21^ ^]^

(2) $$\Delta \phi_{N,G}=\int_{0}^{L}\frac{1}{k_{N}}\left\{\langle k_{Nx}^{2}\rangle +\langle k_{Ny}^{2}\rangle \right\}dz$$

where *k_Nx_* and *k_Ny_* are wave‐vector components along *x* and *y* directions, respectively; < > denotes the average operation. The lasing frequency of a transverse mode can be obtained from Equation (1)

(3) $$v_{N}=\frac{c}{2[n_{\mathrm{cell}}h+n_{e}\left(L-h\right)]}\left(q+\frac{1}{\pi }\Delta \phi_{N,G}\right)$$

In Equation (3), *n*_cell_ is the effective RI of the region where the laser overlaps with a cell, *n*_e_ is the effective RI of the surrounding environment, and *h* is the cell height. Assuming that a transverse mode satisfies the paraxial approximation, the lasing frequency can be expressed as:

(4) $$v_{N}=\frac{c}{2[n_{\mathrm{cell}}h+n_{e}\left(L-h\right)]}\left[q+\frac{1}{\pi }\left(N+1\right)\Delta \phi_{0,G}\right]$$

Subsequently, the transverse‐mode frequency spacing can be obtained:

(5) $$\Delta \nu =v_{N+1}-v_{N}=\frac{c}{2\pi [n_{\mathrm{cell}}h+n_{e}\left(L-h\right)]}\Delta \phi_{0,G}$$

Here Δ*ϕ*_0,_ *_G_* is the Gouy phase shift of fundamental transverse mode, which can be calculated based on ABCD matrix analysis:

(6) $$\begin{array}{ccc} \Delta \phi_{0,G} & = & \arctan\left[\frac{h\sqrt{4-{\left(A_{0}+D_{0}\right)}^{2}}}{2B_{0}}\right]\\ & & +\arctan\left[\frac{\left(L-h\right)\sqrt{4-{\left(A_{1}+D_{1}\right)}^{2}}}{2B_{1}}\right] \end{array}$$

*A*_0_ (*A*_1_), *B*_0_ (*B*_1_), and *D*_0_ (*D*_1_) are elements of round‐trip *ABCD* matrices, which can be obtained by:

(7) $$\begin{array}{c} \left[\begin{array}{c} A_{0}\\ C_{0} \end{array}\right.\;\left.\begin{array}{c} B_{0}\\ D_{0} \end{array}\right]=\left[\begin{array}{c} 1\\ 0 \end{array}\right.\;\left.\begin{array}{c} h\\ 1 \end{array}\right]\left[\begin{array}{c} 1\\ \frac{n_{e}-n_{\mathrm{cell}}}{n_{\mathrm{cell}}R} \end{array}\right.\;\left.\begin{array}{c} 0\\ \frac{n_{e}}{n_{\mathrm{cell}}} \end{array}\right]\left[\begin{array}{c} 1\\ 0 \end{array}\right.\;\left.\begin{array}{c} L-h\\ 1 \end{array}\right]\left[\begin{array}{c} 1\\ 0 \end{array}\right.\;\left.\begin{array}{c} L-h\\ 1 \end{array}\right]\left[\begin{array}{c} 1\\ \frac{n_{e}-n_{\mathrm{cell}}}{n_{e}R} \end{array}\right.\;\left.\begin{array}{c} 0\\ \frac{n_{\mathrm{cell}}}{n_{e}} \end{array}\right]\left[\begin{array}{c} 1\\ 0 \end{array}\right.\;\left.\begin{array}{c} h\\ 1 \end{array}\right]\\ \left[\begin{array}{c} A_{1}\\ C_{1} \end{array}\right.\;\left.\begin{array}{c} B_{1}\\ D_{1} \end{array}\right]=\left[\begin{array}{c} 1\\ 0 \end{array}\right.\;\left.\begin{array}{c} L-h\\ 1 \end{array}\right]\left[\begin{array}{c} 1\\ \frac{n_{e}-n_{\mathrm{cell}}}{n_{e}R} \end{array}\right.\;\left.\begin{array}{c} 0\\ \frac{n_{\mathrm{cell}}}{n_{e}} \end{array}\right]\left[\begin{array}{c} 1\\ 0 \end{array}\right.\;\left.\begin{array}{c} h\\ 1 \end{array}\right]\left[\begin{array}{c} 1\\ 0 \end{array}\right.\;\left.\begin{array}{c} h\\ 1 \end{array}\right]\left[\begin{array}{c} 1\\ \frac{n_{e}-n_{\mathrm{cell}}}{n_{\mathrm{cell}}R} \end{array}\right.\;\left.\begin{array}{c} 0\\ \frac{n_{e}}{n_{\mathrm{cell}}} \end{array}\right]\left[\begin{array}{c} 1\\ 0 \end{array}\right.\;\left.\begin{array}{c} L-h\\ 1 \end{array}\right] \end{array}$$

In Equation (7), *R* is the curvature radius of a cell at the top surface, which can be utilized to characterize the local cell morphology. Substituting Equations (6) and (7) into Equation (5), we calculated the transverse‐mode frequency spacing as a function of curvature radius of a cell. Here we only consider the frequency spacing between the first‐order (TEM_01_) and fundamental modes (TEM_00_), since these two modes are much closer to the paraxial approximation due to the small spot sizes. As shown in Figure 1c,d, the transverse‐mode frequency spacing is highly correlated to the cell curvature, which decreases dramatically with the increasing local curvature radius. Therefore, the transverse‐mode frequency spacing can be utilized to record the local morphology of a cell.

We further performed the simulations to show how the cell morphology in a larger area affects the frequency distribution of transverse modes with increasing orders. Various cell morphologies were exemplified and investigated in Figure 1e–g, including cells with small, large, and uneven local surface curvatures (more common in real cells). The frequency distributions extracted from Figure 1e–g are plotted in Figure 1h, where case 1 and case 2 increase almost linearly with the increasing mode orders. Note that the lasing frequencies increase slower with the mode orders for the cell with a smaller curvature, resulting in narrower distribution of laser modes in the hyperspectral image (right panel in Figure 1f). For a cell with an uneven surface and larger local curvature (Figure 1g), the lasing frequencies increase “nonlinearly” with the mode orders (case 3 in Figure 1h). A non‐uniform distribution of laser modes will be observed in the hyperspectral image (right in Figure 1g). To have a better interpretation, we employed the average frequency spacing of a transverse mode [Δ*ν*_ave_ = (*v_N_* − *v*_0_)/*N*] to characterize different cell morphologies in Figure 1i. For cells with smooth profiles, the average frequency spacings remain constant for individual cells. For a cell with an uneven profile, the average frequency spacings decrease with the mode orders. The simulations demonstrate that the cell morphology strongly governs the frequency spacings of transverse modes. As such, the frequency distribution of laser modes can serve as the mapping of a cell.

### 2.2 Lasing from Cell Models

**Figure 2:**
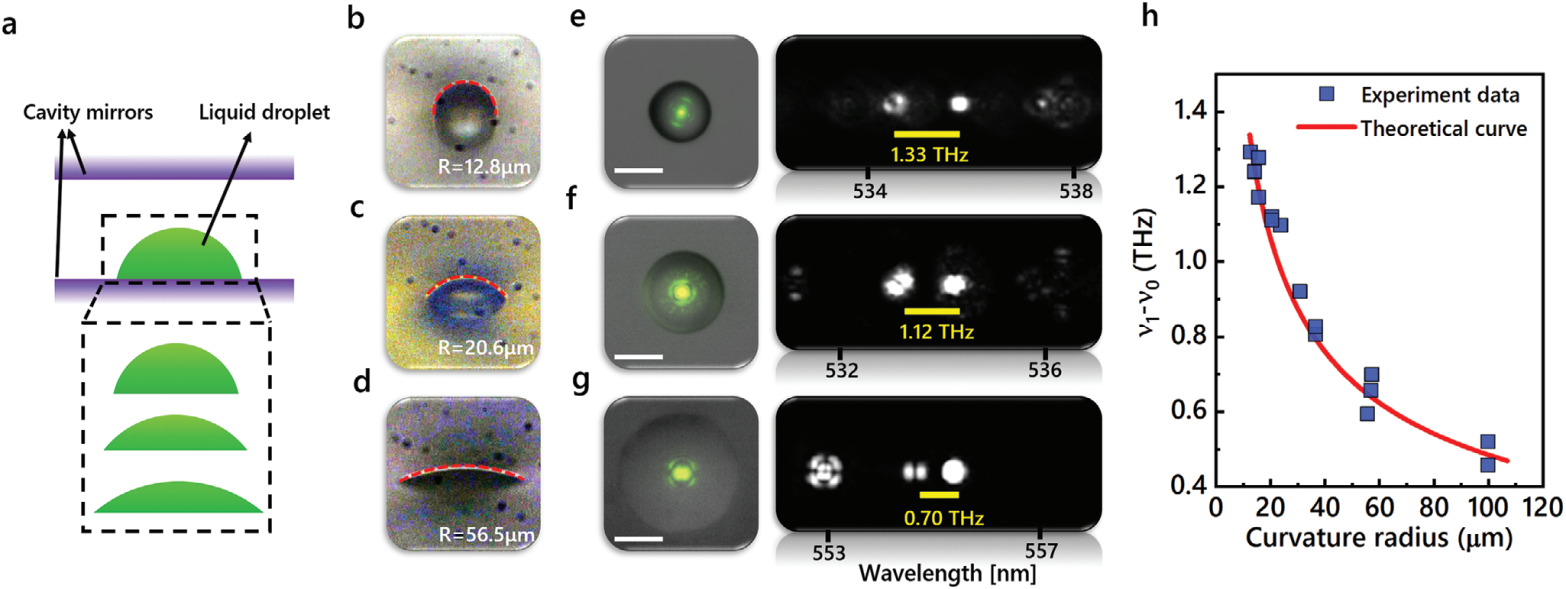
Laser modes from droplets with different curvature radii. a) Schematic of FP cavity sandwiching water/glycerol droplets with different curvature radii (simulating cell adhesion process). b–d) Side‐view of droplets with different curvature radii. *R*, curvature radius. e–g) Far‐field laser emission patterns from droplets superimposed on the bright‐field images (left), and the hyperspectral images of laser modes (right), with respect to (b–d). Yellow lines denote the wavelength spacing between TEM_01_ and TEM_00_ modes. h) Frequency spacing between TEM_01_ and TEM_00_ modes as a function of droplet curvature radius. Scale bar: 20 µm.

To begin with, liquid droplets formed by water/glycerol were first fabricated in FP cavities to show how the cross‐sectional curvature of a droplet affects the transverse‐mode frequency spacing. By using mirrors with different hydrophobicity, dye‐doped droplets with different curvatures were formed, as shown in **Figure** **2**a. Subsequently, we measured the frequency spacings between the first‐order modes (TEM_01_) and fundamental modes (TEM_00_) based on droplets with different curvatures. Figure 2b–d illustrates the side‐view profiles of three individual droplets with different curvature radii. Meanwhile, the corresponding laser emission patterns and hyperspectral images of transverse modes are presented in Figure 2e–g. By comparing the hyperspectral images of transverse modes (right panel in Figure 2e–g), the frequency spacing decreases from 1.33 to 0.70 THz as the droplet curvature radius increases from 12.8 to 56.5 µm (Figure 2b–d). In Figure 2h, we compared the experimental data with theoretical results based on Equation (5). The measured frequency spacings as a function of droplet curvature radius (blue dots) are in good agreement with the theoretical curve (red line). It is worth noting that, in addition to round droplets shown in Figure 2, an elliptical droplet was also investigated by laser modes, for which the local curvatures along two vertical directions can be distinguished by two orthogonal first‐order modes (HG_01_ and HG_10_ modes in Figure S2, Supporting Information).

### 2.3 Lasing Analysis from Biological Cells

**Figure 3:**
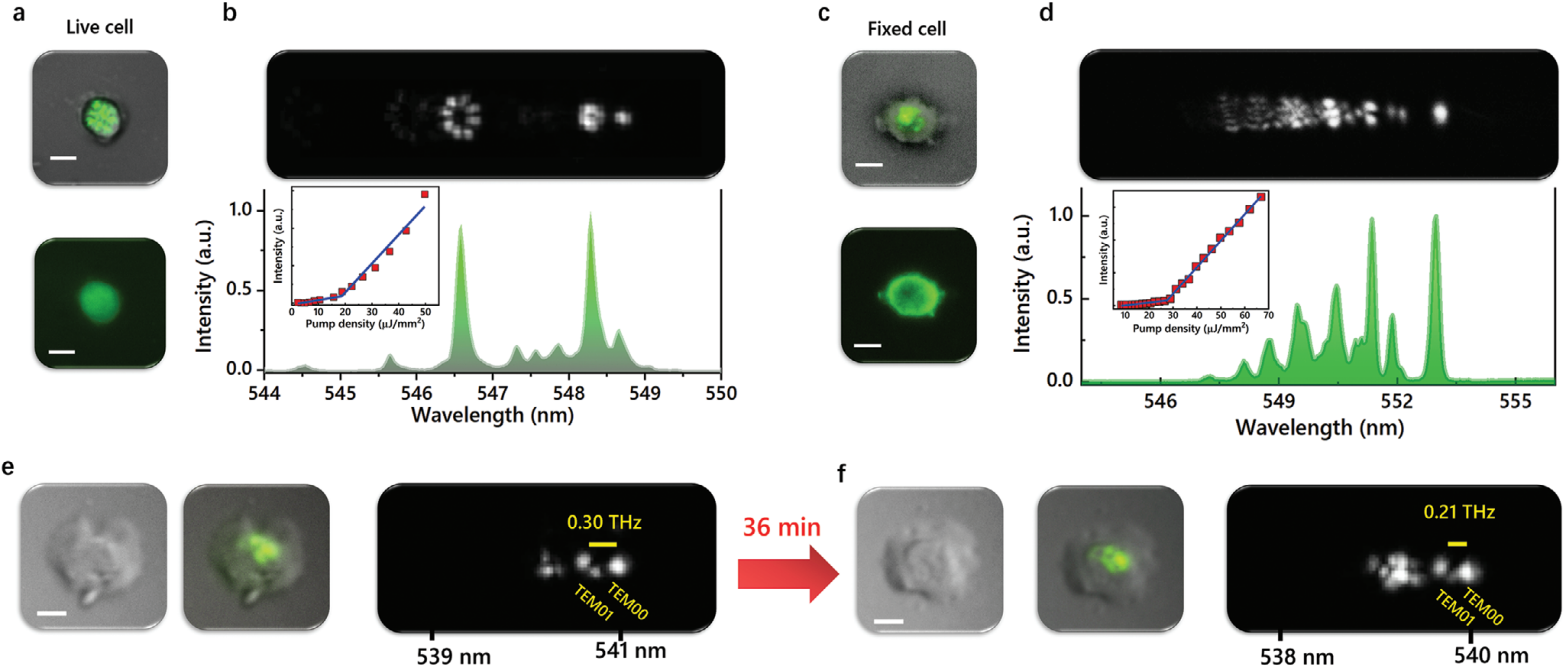
Lasing from live and fixed C2C12 cells. a) Far‐field laser emission pattern (top) and fluorescence image (below) of a live C2C12 cell stained with CMFDA. b) Hyperspectral imaging of laser modes and the corresponding lasing spectrum of live C2C12 cell in (a). Insert: spectrally integrated output intensities as a function of pump energy density. c) Far‐field laser emission pattern (top) and fluorescence image (below) of a fixed C2C12 cell stained with FITC‐NHS. d) Hyperspectral imaging of laser modes and the corresponding lasing spectrum of fixed C2C12 cell in (c). Insert: spectrally integrated output intensities as a function of pump energy density. e‐f) Bright‐field images of a live C2C12 (left), far‐field laser emission pattern (middle) and the corresponding hyperspectral images of laser modes (right) recorded e) before and f) after 36 min. Scale bar: 10 µm

Next, we demonstrated laser emissions from cultured biological cells, in which both live and fixed C2C12 cells were studied. The C2C12 cells were cultured directly on a highly reflective dielectric mirror and measured under different time periods. 5‐chloromethylfluorescein diacetate (CMFDA) and 5/6‐carboxyfluorescein succinimidyl ester (FITC‐NHS) were exploited as the fluorescent dye for live and fixed cells, respectively. These two probes were selected based on similar emission wavelengths. By covering the top mirror to form a FP cavity, lasing actions from live and fixed cells were achieved upon excitation, as presented in **Figure** **3**a–d. The far‐field laser emission patterns were formed by the superposition of a series of transverse modes in Figure 3a (live cell) and Figure 3c (fixed cell), respectively. The hyperspectral images in Figure 3b,d present the patterns of transverse modes, where the corresponding lasing spectra and wavelengths are shown below. To further verify lasing operations, spectrally integrated output intensities as a function of pump energy density were also measured (insets in Figure 3b,d), which shows clear threshold behaviors of the cell lasers.

In general, the curvature of a live cell changes through time as it adheres to the cultured surface. Hence, we monitored the evolution of transverse modes from a cell inside the microscopic incubator for a period of time. As shown in Figure 3e,f, a decrement in frequency spacing between TEM_01_ and TEM_00_ modes were observed in hyperspectral images after 36 min. This result indicates a decreasing local curvature on the surface membrane of the cell, which, however, was relatively difficult to observe in the bright‐field images (left panel of Figure 3e,f). Our findings demonstrate that frequency spacings of transverse modes possess high sensitivity to cell morphology. More examples of laser output from live cells are shown in Figure S3, Supporting Information.

**Figure 4:**
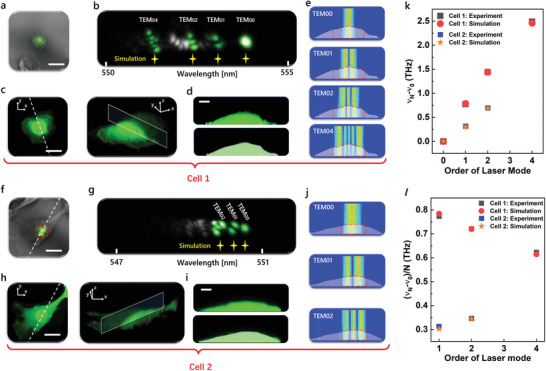
Cell characterization with laser modes and confocal fluorescence microscopy. a) Far‐field laser emission patterns superimposed on bright‐field images. b) Hyperspectral images of laser modes. Laser modes with the same orientation were selected to perform mode simulation (labeled in green). Yellow stars represent the relative wavelength‐positions of laser modes based on the frequency spacings obtained from simulations. c) Confocal microscopic images of cell 1 and its 3D cross‐section plane. d) Cell profiles from the cross‐section plane in (c). Top: confocal microscopic images. Bottom: cell profiles used in the simulations superimposed on the confocal microscopic images. e) Simulations of laser modes oscillations in FP cavity for cell 1. Red dashed lines denote the practical region covered by laser modes. f) Far‐field laser emission patterns superimposed on bright‐field images. Red dashed circle: cell nucleus. g) Hyperspectral images of laser modes. Laser modes selected to perform mode simulation (labeled in green). Yellow stars represent the relative wavelength‐positions of laser modes based on the frequency spacings obtained from simulations. h) Confocal microscopic images of cell 2 and its 3D cross‐section plane. i) Cell profiles from the cross‐section plane in (h). Top: confocal microscopic images. Bottom: cell profiles used in the simulations superimposed on the confocal microscopic images. j) Simulations of laser modes oscillations in FP cavity for cell 2. Red dashed lines denote the practical region covered by laser modes. k) Frequency distributions of laser modes (*ν*_N_ − *ν*_0_) from cell 1 and cell 2. l) Average frequency spacing [(*ν*_N_ − *ν*_0_)/N)] of laser modes from cell 1 and cell 2. Scale bar in (a,c,f,h): 20 µm. Scale bar in (d,i): 5 µm. The two cells were fixed C2C12 cells.

To further analyze the impact of cell morphology on frequency distributions of transverse modes with increasing mode orders, hyperspectral laser images were compared with 3D confocal fluorescence imaging based on identical cells. Experimentally, **Figure** **4** shows two examples of individual cells with different morphologies. By comparing the hyperspectral images in Figure 4b,g, the laser modes from cell‐1 possess larger frequency spacings than cell‐2 due to larger curvature. We selected the TEM_0N_ laser modes with unified long axes for analysis (green marks in Figure 4b,g); in such way, only the effect of cell profile in one cross‐sectional plane needs to be considered. To obtain the cell morphology, 3D confocal scanning of both cells was performed. As shown in Figure 4c,h, we crosscut the 3D confocal image of a cell with a specific plane that intersects with the laser beam along the long‐axis direction of TEM_0N_ modes. Subsequently, the cross‐sectional cell profiles were obtained in Figure 4d,i. As expected, we can clearly observe that cell‐1 has a larger curvature than cell‐2.

We then performed the simulations on the generation of TEM_0N_ modes in FP cavities based on a given cell profile. The cell profiles used in the simulations were highly consistent with the measured ones (below in Figure 4d,i). As shown in Figure 4e,j, the oscillations of TEM_0N_ modes with *N* nodal lines were obtained. The frequency information of TEM_0N_ modes was also extracted and compared with the experimental results, in which *ν_N_* was defined as the frequency of TEM_0N_ mode. The lasing frequency distribution (*ν*_N_ − *ν*_0_) and the average frequency spacing [(*ν*_N_ − *ν*_0_)/*N*] of the transverse modes were both in good agreements with the experiment in Figure 4k,l. In the simulation, the top curvature radii were 4.9 and 43 µm for cell‐1 and cell‐2, respectively, resulting in larger frequency spacings of transverse modes for cell‐1 than cell‐2. In the hyperspectral images (Figure 4b,g), we marked the relative wavelength‐positions of the TEM_0N_ modes based on the frequency spacings obtained from the simulations (yellow stars), which presents nearly the same distribution as the laser modes emitted from the cells. In the simulations, the practical regions overlapped with laser modes in the cells are marked in Figure 4e,j, for which a relatively high RI of 1.41 was assumed by considering the effect of cell nuclei. In the experiments, we noticed that most laser modes were covered by cell nuclei. For instance, the laser modes from cell‐2 were distributed in the area of the nucleus (red dashed outline in Figure 4f). To confirm the effect of a cell nucleus, we measured the 3D confocal microscopic images of adherent C2C12 cells by staining membranes and nuclei with different dyes, as presented in Figure S4, Supporting Information. It can be observed that a nucleus occupies a large volume in a cell, which also strongly governs the cell curvature at the top surface. Therefore, the frequency spacings of laser modes are highly correlated with the cell nucleus.

### 2.4 Recording Cell Adhesion Process by Transverse Modes

**Figure 5:**
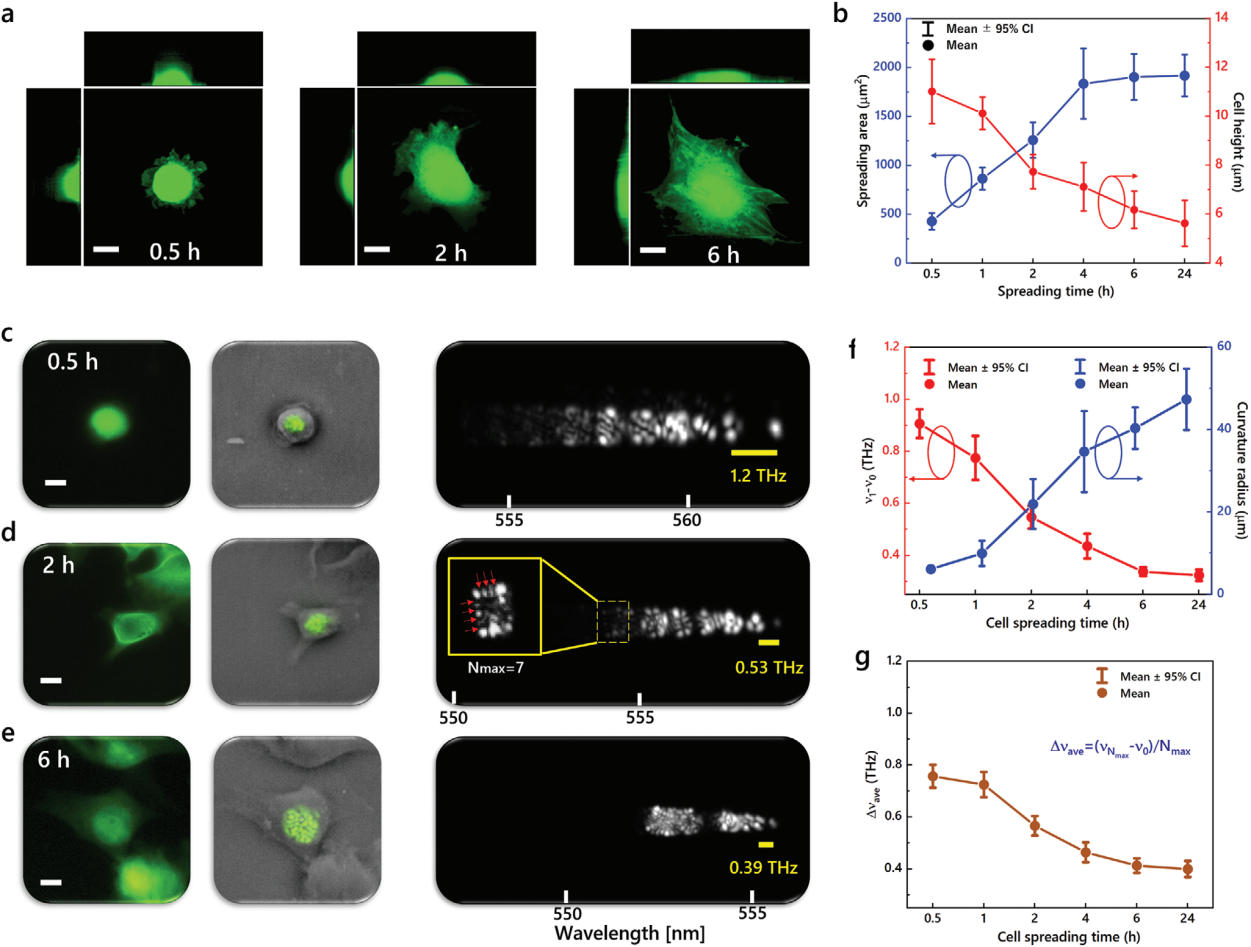
Monitoring cell adhesion process with hyperspectral laser mode imaging. a) Confocal microscopic images of individual cells under spreading time of 0.5, 2, and 6 h. b) Measured spreading areas and cell heights as a function of spreading period. Sample size: *n* = 103 and 149 for measuring cell heights and spreading areas, respectively. c‐e) Fluorescence image (left), far‐field laser emission patterns superimposed on the bright‐field images of the cells (middle) and hyperspectral images of laser modes (right) of three individual cells under spreading periods of c) 0.5, d) 2, and e) 6 h, respectively. Yellow lines denote the wavelength spacing between TEM_01_ and TEM_00_ modes. f) Frequency spacing between TEM_01_ and TEM_00_ modes and estimated cell top curvature radius as a function of cell spreading period. Sample size: *n* = 223. g) Average frequency spacing of laser modes as a function of cell spreading period. Sample size: *n* = 177. Scale bar: 10 µm. All the cells were fixed C2C12 cells.

In principle, cell curvature changes during cell adhesion, which is also highly linked to its nucleus morphology and mechanics. Previous results in Figures 3 and 4 demonstrated that transverse modes can be applied for recording cell spreading behavior by extracting the values of frequency spacings. This triggered us to systematically investigate cell adhesion process with hyperspectral laser images. Herein C2C12 cells with different morphology were investigated, in which the cells were cultured directly on a mirror and fixed after a specific spreading time. We first performed the confocal measurements to confirm the fundamental characteristics (including spreading area and cell height) of targeted cells at a respective spreading time. **Figure** **5**a shows the confocal microscopic images of three individual cells with a spreading time of 0.5, 2, and 6 h, which clearly presents the increment of spreading area and decrement of cell height. In addition, the decrease of local curvatures at the cellular surface can also be observed. For a spreading period of 24 h, the average spreading areas of the cells increased from ≈300 to 2000 µm^2^; meanwhile, the average cell heights decrease from ≈11 to 5.5 µm (Figure 5b). All of the results are typical characteristics of cell spreading with time.^[^ ^22^ ^]^

Single‐cell laser experiments were then conducted to record the frequency spacings of transverse modes based on cells with different cell spreading time. Figure 5c–e presents the fluorescence images and laser mode images of three individual cells with a spreading time of 0.5, 2, and 6 h, respectively. In the hyperspectral images, a narrower distribution of laser modes was found for cells with longer spreading time. The decrement of frequency spacing between TEM_01_ and TEM_00_ modes can be clearly observed (yellow lines in Figure 5c–e). The statistical results in Figure 5f show that the frequency spacings between TEM_01_ and TEM_00_ modes decreased from 0.9 to 0.3 THz as the spreading time increased from 0.5 to 24 h. According to Equation 5, the local curvature radii of the cells were estimated to increase from 5 to 48 µm based on theoretical calculation. We also measured the average frequency spacing of highest‐order transverse modes, which is defined by Δ*ν*_ave_ = (*v_N_*_max_ − *v*_0_)/*N*_max_ (*N*_max_: the highest order of the laser mode generated). Here, the order of a laser mode equals the number nodal lines in a laser pattern. For example, the highest‐order of laser mode in the hyperspectral image possesses seven nodal lines in Figure 5d; thus, the highest order is 7. The statistical results of average frequency spacings also show a decreasing trend with the increasing spreading time (Figure 5g). Note that the highest‐order laser mode covers a larger area of a cell. As such, the average transverse‐mode frequency spacing can reflect an “average” cell curvature in a larger area. In comparison between Figure 5f and Figure 5g, we noticed that the average frequency spacing of highest‐order modes is smaller than the frequency spacing between TEM_01_ and TEM_00_ modes at a shorter spreading time, for example, 0.5 h. This phenomenon indicates that the curvature of a cell is larger at the top‐surface of cell but becomes smaller at the cell waist. Such morphological characteristics can be referred to Figures 1g and 4d, for which a higher‐order transverse mode possesses a smaller average frequency spacing. In principle, the transverse‐mode frequency spacing is highly correlated to the lens effect provided by a cell. A larger cell curvature and a higher RI will both cause a larger frequency spacing. Under a fixed cell curvature and RI, the cell height contributes less to the lens effect in a cavity. As demonstrated in Figure S5, Supporting Information, the frequency spacing is not sensitive to the cell height. We also calculated the frequency spacings under different RI in Figure S5, Supporting Information, where obvious differences could be observed under various RI values. However, note that all the laser data in Figures 4 and 5 were measured based on the same type of cells (similar RI). Consequently, the changes of frequency spacings should be dominated by the cell curvatures rather than the RI of cells.

### 2.5 Cell Classification by Using Transverse Modes

**Figure 6:**
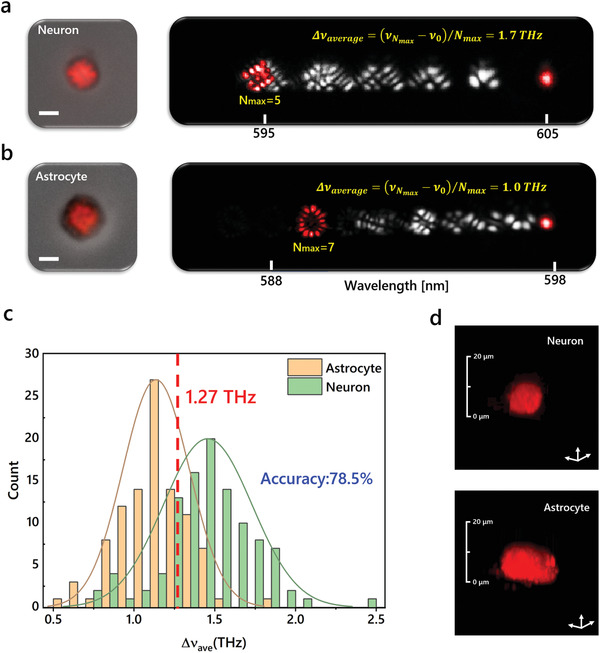
Classification of cortical neurons and astrocytes using laser modes. a) Far‐field laser pattern of a cortical neuron superimposed on the bright‐field image (left) and the hyperspectral image of laser modes (right). Red labeled laser modes correspond to the fundamental modes and highest‐order modes. b) Far‐field laser pattern of an astrocyte superimposed on the bright‐field image (left) and the hyperspectral image of laser modes (right). c) Statistics of average frequency spacings of laser modes from cortical neurons and astrocytes. Sample size: *n* = 105 and 100 for neurons and astrocytes, respectively. d) Confocal fluorescence microscopic images of a cortical neuron and an astrocyte labeled with Rh6G. Scale bar: 5 µm.

We finally explored the possibility of employing transverse modes for cell classification, in which fixed cortical neurons and astrocytes extracted from rats were investigated. Instead of using adhesion cells, neurons and astrocytes were suspended for testing if laser modes could identify different cell types even in suspension (**Figure** **6**). The cells were stained with Rhodamine 6G and sandwiched in FP cavities, forming single cell lasers. Hyperspectral images of laser modes were recorded based on a fixed pump energy. We utilized the average frequency spacing of the highest‐order transverse modes Δ*ν*_ave_ as the key parameter for cell classification. This is because the highest order of laser mode covers a larger area of a cell, which can reflect a global morphological characteristic of each cell. As shown in Figure 6a,b, we noticed that the laser modes from a cortical neuron possess larger frequency spacings than those from an astrocyte. The average transverse‐mode frequency spacing is 1.7 THz for the cortical neuron but is only 1.0 THz for the astrocyte. We performed the statistics to investigate the classification accuracy, in which ≈100 cells were randomly selected from each cell type. Figure 6c illustrates the distributions of average frequency spacings Δ*ν*_ave_ for cortical neurons and astrocytes. The Gaussian distribution fittings present 0.3 THz difference between the central values of Δ*ν*_ave_ for cortical neurons and astrocytes. By choosing 1.27 THz as the classification criterion, the classification accuracy reached 78.5%. The difference in terms of frequency spacings originated from different morphological characteristics of cortical neurons and astrocytes. As compared in Figure 6d, the confocal microscopic images show that a cortical neuron has a larger curvature than an astrocyte in the side‐view. It is worth noting that, we also recorded the frequency spacings between TEM_01_ and TEM_00_ modes as the parameter for the classification of cortical neurons and astrocytes (Figure S6, Supporting Information). However, the classification accuracy was only 72%, indicating that the local curvature of a cell is not a better feature for the classification. Overall, the results here also present the potential of cell lasers for the classification of cell populations with different morphological features by extracting the frequency information of transverse modes.

## 3 Discussion and Conclusion

To verify the repeatability of the present experimental setup, we evaluated the effect of slight cavity variations on the frequency spacings of laser modes. Considering that the cavity lengths vary in 2 µm‐range and the tilting angles of top mirrors vary in 1̊‐range, our results show that only slight changes in the frequency spacings were observed (Figure S7, Supporting Information). We also measured the frequency spacings under different FP cavities based on the same spacers repeatedly, in which liquid hemisphere droplets were employed as cell models (Figure S8, Supporting Information). The frequency spacings fluctuated in a small range under different FP cavities and locations, presenting a good repeatability. For all the laser experiments in this work, we carefully spread microbeads to cover a large area of a mirror as spacers. Meanwhile, magnets were used to sandwich the mirrors for fixing a cavity. The purpose of all the operations is to minimize the cavity variations. In the future, we may further minimize experimental errors by fabricating an optofluidic microlaser device through 3D printing. In such frame, high‐throughput cell analysis may also be performed easily.

The emission of transverse modes is an inherent property of FP cell lasers; therefore, the distributions of spatial intensities and wavelengths of transverse modes can be regarded as a distinct fingerprint of each cell. In principle, one can reveal the correlation between the attributes of transverse modes (patterns and wavelengths) and the 3D spatial property of a cell (3D morphology and RI distribution) by numerically solving Helmholtz equation based on 3D model. However, a huge amount of calculation is required, which is the main limitation for practical applications of cellular lasers. This study proposed a simple method for cell analysis based on transverse modes by extracting useful frequency information. By using the frequency spacing between lowest‐order laser modes, the local curvature of a cell can be recorded. Considering that the local curvature of a cell is related to the surface tension, laser modes can be applied for studying cell mechanics.^[^ ^23^, ^24^ ^]^ Apart from the local cell curvatures as discussed in Figure 4, the 3D local curvatures of a cell can also be recorded by transverse modes (Figure S9, Supporting Information). Due to the fact that a cell nucleus mainly contributes to laser oscillations, frequency spacings can be used for studying the deformation of a nucleus which is induced by the change of nuclear mechanics.^[^ ^24^, ^25^, ^26^ ^]^ In addition to local morphology, the morphological characteristics in a larger area can be recorded by average frequency spacings of laser modes defined in Figure 6. In such a way, cell classification or phenotyping can be realized by employing laser modes. Due to the fingerprint of transverse modes, the classification accuracy can be further improved by learning the hyperspectral images of laser modes with artificial intelligence technologies,^[^ ^27^ ^]^ in which all the information of laser modes would be applied, including the patterns and frequency distributions. The similar approach can be expanded to wider cell populations with different morphological characteristics.

Many powerful techniques, such as atomic force microscopy and quantitative phase imaging, have been developed for studying cell morphology.^[^ ^28^, ^29^ ^]^ However, cell laser that takes advantage of cavity‐resonant principles possesses their unique characteristics of high sensitivity, with potentials for sensing and probing subtle dynamic changes in cells. Other resonator‐based techniques have also been proposed for recording the spreading behaviors of adherent cells,^[^ ^30^, ^31^, ^32^, ^33^ ^]^ which focused on studying the mechanics at the adherent interface. In contrast, FP cell lasers can reflect the local and global morphology changes of adherent cells during spreading. In current experimental conditions, FP cavities are less suitable for long‐term live cell experiments. In the future, we may improve the nutrient supplements for live cells by integrating a microfluidic system or microwells within a FP cavity. With the design of microwell FP cavity, each cell can be placed within a single well with larger volume, where more nutrients can be delivered to individual cell. By removing the top mirror, cell supplements can be exchanged with appropriate designs provided that the cavity is stable enough. In addition, high‐throughput measurements with large areas could be realized by employing a scanning system.

In conclusion, we have revealed the basic principle of how a single cell governs the lasing frequencies of transverse modes from cell lasers. Cellular features and bioinformation which a cellular laser can potentially provide were demonstrated. Theoretical studies were conducted to demonstrate that transverse‐mode frequency spacing is highly correlated with cell curvature and morphology. A smaller local curvature of a cell shortens the frequency spacing between TEM_01_ and TEM_00_ modes; meanwhile, the whole profile of a cell determines the frequency distribution of laser modes with increasing orders. Experimentally, the spectral characteristics of transverse modes were investigated in live and fixed cells with different morphological features. By extracting the frequency information, transverse modes were utilized for studying cell adhesion process and cell classification. This study represents a milestone in biological cell lasers, opening new possibilities in biophotonic applications by means of hyperspectral laser imaging.

## 4 Experimental Section

### Materials

Poly‐d‐Lysine (PDL) (P0899), DNase 1 type IV, Triton X‐100, FITC‐NHS, Rhodamine 6G (Rh6G), fluorescein sodium salt (FITC), and 1,1′‐Dioctadecyl‐3,3,3′,3′‐tetramethylindocarbocyanine perchlorate were purchased from Sigma‐Aldrich. Alexa‐Fluor 488 goat anti‐mouse, 4′,6‐diamidino‐2‐phenylindole, paraformaldehyde (PFA, 7 230 681), goat serum, Dulbecco's modified eagle medium (DMEM), penicillin/streptomycin (Pen/Strep), B27 supplement (17 504 001), GlutaMAX, neurobasal medium, Fluoromount‐GTM mounting medium (00‐4958‐02), fetal bovine serum (FBS), CMFDA, and YO‐PRO‐1 iodide were purchased from Life Technologies. Mouse anti‐Tuj1 antibody (801 202) was purchased from Biolegend. Rabbit anti‐GFAP (Z0334) was obtained from DAKO. Papain suspension was purchased from Worthington. Cell strainer (70 µm) was obtained from BD, Biosciences, USA.

### Preparation of Water‐Based Droplets

Mixture of DI water/glycerol (volume ratio of 1:1) doped with 2 mm FITC was used as the droplet material in Figure 2. An inkjet printer was used to print the mixture on dielectric mirrors with different hydrophobicity, forming droplets with different curvatures as well as contact angles. The hydrophobicity of the mirrors was tuned by treating the surfaces with different ratios of hydrophobic reagent/ethanol solutions. The droplets possess a fixed volume of 4.4 pL.

### Acquisition of C2C12 Cells

The mouse skeletal myoblast cell line C2C12 was cultured in DMEM supplemented with 10% FBS and 1% Pen/Strep. The cells were grown on a standard T75 cell culture flask in a humidified incubator at 37 °C and 5% CO_2_, and were subcultured at 70–80% confluence to avoid induction of differentiation. The growth medium was changed every other day. For the fixed C2C12 cells used in laser experiments, we first seeded live cells onto six identical mirrors and cultured for 0.5, 1, 2, 4, 6, and 24 h, respectively. The samples were then washed with PBS and fixed with 4% PFA for 20 min at room temperature. Thereafter, the samples were washed twice with PBS. The fixed C2C12 cells were stained by immersing the mirrors in 500 µm FITC‐NHS/PBS solutions for 15 min, and were finally washed with PBS for the further laser experiments. For the live C2C12 cells used in laser experiments, the cells were directly cultured on a mirror immersed in serum‐free medium containing 100 µm CMFDA and 2% DMSO.

### Acquisition of Cortical Neurons and Astrocytes

The animal care and experimental procedures were carried out in accordance with the Institutional Animal Care and Use Committee guidelines of Nanyang Technological University (IACUC, NTU, Protocol number A19004). Briefly, P0‐P2 neonatal rat cortices were isolated and digested with 1.2 U papain and 40 µg mL^−1^ of DNase at 37 ℃ for 1 h. Thereafter, the digestion process was stopped by adding 8 mL of DMEM with 10% FBS. For the acquisition of cortical neurons, a portion of the dissociated tissue was gently triturated with 1 mL pipette for 11 times. The cell suspension was passed through a 70 µm cell strainer to obtain the dissociated cortical neurons.

For the acquisition of astrocytes, a further culture step was necessary in order to allow them to proliferate before collection. To achieve that, the remaining unused dissociated tissues were triturated with a syringe connected with a 21G needle, followed by a 23G needle for ten times each. The triturated cell suspension was then seeded on four PDL‐coated (100 µg mL^−1^) T75 flasks and cultured in DMEM with 10% FBS and 1% Pen/Strep for further separation of glial cells. The medium was changed every three days.

After 9–11 days of culture, the four T75 flasks were placed on an orbital shaker and shaken (200 rpm, 37 °C) for 17–19 h to remove the microglial and oligodendrocyte progenitor cells. The remaining cells on the flasks were trypsinized with 2 mL of 0.25% trypsin‐EDTA at 37 ℃ for 5 min to obtain the astrocytes. The enzymatic activity was stopped by adding 8 mL of DMEM containing 10% FBS. The cells were then spun down at 300 g for 5 min. Thereafter, the astrocytes were resuspended in 3 mL of DMEM containing 10% FBS and 1% Pen/Strep and triturated with a 1 mL pipette for ten times before they were passed through a 70 µm cell strainer. Antibody labeling was performed to check the purity of the dissociated neurons and astrocytes (Figure S10, Supporting Information).

For the cell laser experiment, the collected cortical neurons and astrocytes were spun down at 300 g for 5 min to discard the supernatant. The cells were then fixed with 4% PFA for 20 min before they were spun down (300 g, 5 min) and resuspended in 1× PBS for subsequent usage. Rh6G dye was used to stain the fixed cells. 0.48 mg Rh6G powder was dissolved by 500 µL alcohol and diluted by 500 µL DI water. Thus, the Rh6G concentration of the final solution is 2 mm. The fixed cells were finally stained by adding the Rh6G solution in the PBS solution storing fixed cells with the volume ratio of 1:1.

### Optical System Setup

The schematic of optical system can be referred to Figure 1a and Figure S1, Supporting Information. The cavity mirrors used for droplets and C2C12 cells possessed a high‐reflectivity in the spectral band from 500 to 580 nm; and the cavity mirrors used for cortical neurons and astrocytes possessed a high‐reflectivity in the spectral band from 570 to 710 nm. Microbeads with 27‐ and 16‐µm diameters were used to fix the cavity lengths for droplets and biological cells, respectively. The pump source was a pulsed ns‐laser (EKSPLA NT230) integrated with an optical parametric oscillator (repetition rate: 50 Hz; pulse duration: 5 ns). An upright microscopic system (Nikon Ni2) with 10 × 0.3 NA objective was used to focus the pump light onto a cell, and was also used to collect the emitted laser. The pump beam diameter at the objective focal plane was measured to be 40 µm. For C2C12 cells, the pump wavelength was 480 nm, with the pump energy density of 68 µJ mm^−2^. For cortical neurons and astrocytes, the pump wavelength was 532 nm, with the pump energy density of 149 µJ mm^−2^. The collected laser emissions from a FP cavity were separated by a beam splitter and incident into a charge‐coupled device camera (Newton 970 EMCCD) and imaging spectrometer (Andor Kymera 328i). Nikon A1+ confocal laser microscope system was used for the confocal microscopy measurements, in which a 100 × 1.30 NA oil objective was utilized.

### Simulations and Calculations

The oscillations of laser modes from FP cavities were simulated with the finite element method using Comsol Multiphysics software. The Eigen frequency study was applied in the electromagnet waves, frequency domain interface within the wave optics modules, in which 2D model was employed. By considering the effects of cell nuclei, the RI of the region where the laser overlapped with a cell was assumed as 1.41 for all the calculations on biological cell lasers.^[^ ^34^ ^]^ Moreover, the surrounding environment of cells was set as 1.334; and the cavity length was 16 µm. In Figure 1e,f, the cell curvatures were 8 and 70 µm, respectively. In Figure 1g, the local curvature at the center was 4 µm and the curvature at the waist was 70 µm. In Figures 1d and 5f, the fixed cell height was used as 8 µm for the calculations. In Figure 2h, the RI of droplets and the surrounding environment were 1.41 and 1, respectively; the cavity length was 27 µm; and the droplet volume was 4.4 pL.

### Statistical Analysis

In Figure 4 only two cells were used for proof‐of‐concept. In Figure 5b, 103 cells were selected for measuring the cell heights, in which the numbers of cells for the spreading time periods of 0.5, 1, 2, 4, 6, and 24 h were 18, 18, 22, 14, 14, and 17, respectively. 149 cells were selected for measuring the spreading areas, in which the numbers of cells for the spreading time periods of 0.5, 1, 2, 4, 6, and 24 h were 32, 23, 33, 21, 28, and 12, respectively. In Figure 5f, 223 cells were selected for measuring the transverse‐mode frequency spacings, in which the numbers of cells for the spreading time periods of 0.5, 1, 2, 4, 6, and 24 h were 46, 21, 44, 32, 25, and 55, respectively. In Figure 5g, 177 cells were selected for measuring the average frequency spacings, in which the numbers of cells for the spreading time periods of 0.5, 1, 2, 4, 6, and 24 h were 22, 17, 39, 30, 24, and 45, respectively. In Figure 6, 105 neurons and 100 astrocytes were studied for cell classification. The Gaussian fittings were performed based on Origin Software. Data presentation in Figure 5b,f,g: mean ± 95% CI.

## Conflict of Interest

The authors declare no conflict of interest.

## Author Contribution

Z.Q., Y.‐C.C., and S.Y.C. conceived and designed the experiments; Z.Q. performed the optical experiments; N.Z. conducted the animal experiments; H.X., G.Y., C.G., and X.G. conducted the cellular preparation and experiments. Z.Q. performed the theoretical calculations; Z.Q., S.Y.C., and Y.‐C.C. analyzed the data and wrote the paper. Y.‐C.C. and C.H. supervised the whole research.

## Supporting information

SupportingInformation is available from the Wiley Online Library or from the author.

## Data Availability

The data that support the findings of this study are available from the corresponding author upon reasonable request.
