# Supplementary material for: Cellular Features Revealed by Transverse Laser Modes in Frequency Domain
Source: Adv Sci (Weinh). 2021 Nov 28;9(1):2103550. doi: 10.1002/advs.202103550 (PMC8728842; doi:10.1002/advs.202103550)
Supplement: Supplementary file 1 — SupportingInformation is available from the Wiley Online Library or from the author. [file ADVS-9-2103550-s001.pdf]

ADVANCED SCIENCE
Open Access

Supporting Information

for *Adv. Sci.*, DOI: 10.1002/advs.202103550

Cellular Features Revealed by Transverse Laser Modes in Frequency Domain

Zhen Qiao, Hongmei Xu, Na Zhang, Xuerui Gong, Chaoyang Gong, Guang Yang, Sing Yian Chew, Changjin Huang, Yu-Cheng Chen,**

Supporting Information

Cellular Features Revealed by Transverse Laser Modes in Frequency Domain

Zhen Qiao[1], Hongmei Xu[2], Na Zhang[3], Xuerui Gong[1], Chaoyang Gong[1], Guang Yang[1], Sing Yian Chew[3, 4], Changjin Huang[2, *], Yu-Cheng Chen[1, 3 *]

[1] School of Electrical and Electronics Engineering, Nanyang Technological University, 50 Nanyang Ave., Singapore 639798, Singapore

[2] School of Mechanical and Aerospace Engineering, Nanyang Technological University, 50 Nanyang Ave., 639798, Singapore

[3] School of Chemical and Biomedical Engineering, Nanyang Technological University, 62 Nanyang Drive, Singapore 637459, Singapore

[4] Lee Kong Chian School of Medicine, 11 Mandalay Road, Singapore 308232, Singapore

*Correspondence: cjhuang@ntu.edu.sg; yucchen@ntu.edu.sg

1. Immunohistochemical analysis of neurons

The fixed cells were washed with 1 x PBS for 3 times (5 min each) before they were permeabilized in 0.1% Triton X-100 in 1 x PBS for 15 min. Thereafter, the cells were incubated in non-specific blocking solution (5% goat serum) for 1 hour at room temperature, followed by overnight incubation with primary antibody, mouse anti-Tuj1 (1:1000 dilution) at 4 °C. Following that, the cells were washed for three times with 1 x PBS and detected with Alexa Fluor 488 fluorescent secondary antibodies (1:500 dilution) at room temperature for 2 hours. Cell nuclei were stained with 4′,6-diamidino-2-phenylindole (DAPI).

For cell purity check, images were taken using fluorescent microscopy (Leica DMi8). Ten ROIs were imaged and quantified in each experimental repeat. Three experimental repeats were carried out. The purity of the cortical neurons was 80.08 ± 7.68%, which can be seen in Figure S10.

2. Immunohistochemical analysis of astrocytes

The fixed cells were washed with 1 x PBS for 3 times (5 min each) before they were permeabilized in 0.1% Triton X-100 in 1 x PBS for 15 min. Thereafter, the cells were incubated in non-specific blocking solution (5% goat serum) for 1 hour at room temperature, followed by overnight incubation with primary antibody, Rabbit anti-GFAP (1:1000 dilution) at 4 °C. Following that, the cells were washed for three times with 1 x PBS and detected with Alexa Fluor 488 fluorescent secondary antibodies (1:500 dilution) at room temperature for 2 hours. Cell nuclei were stained with 4′,6-diamidino-2-phenylindole (DAPI).

For cell purity check, images were taken using fluorescent microscopy (Leica DMi8). Ten ROIs were imaged and quantified in each experimental repeat. Three experimental repeats were done. The purity of the astrocytes was 95.03 ± 1.90%, which can be seen in Figure S10.

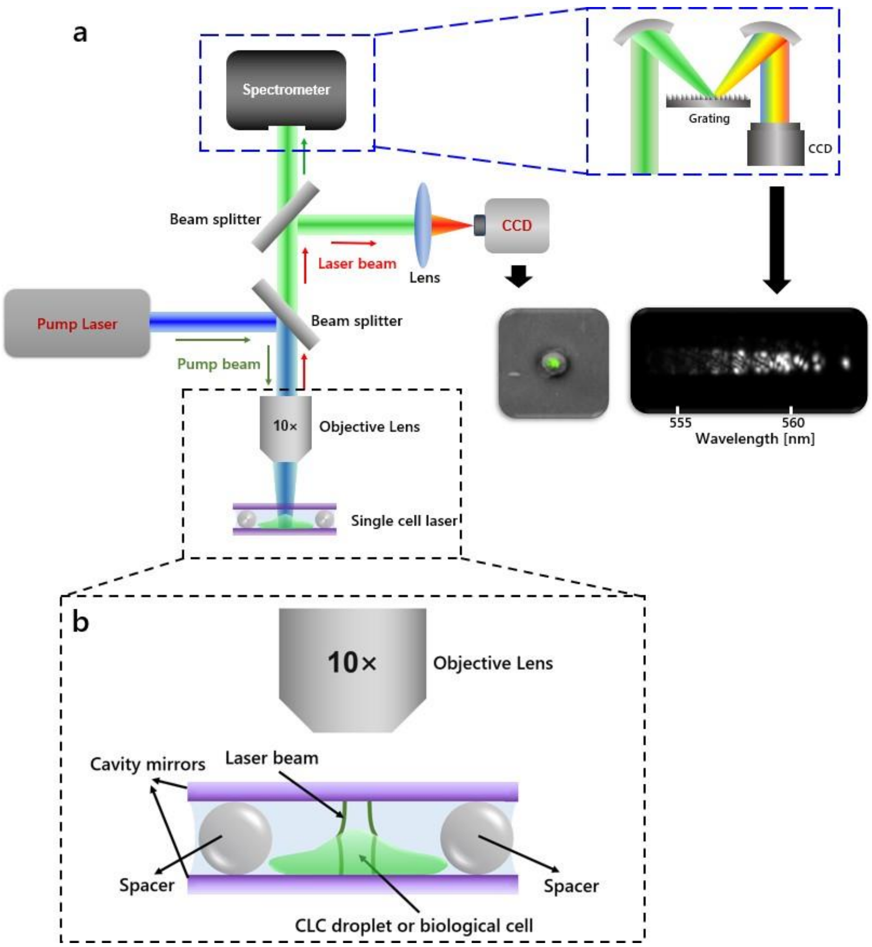

Fig. S1. (a) Schematic of the optical system for single cell laser imaging. (b) Zoomed cell laser.

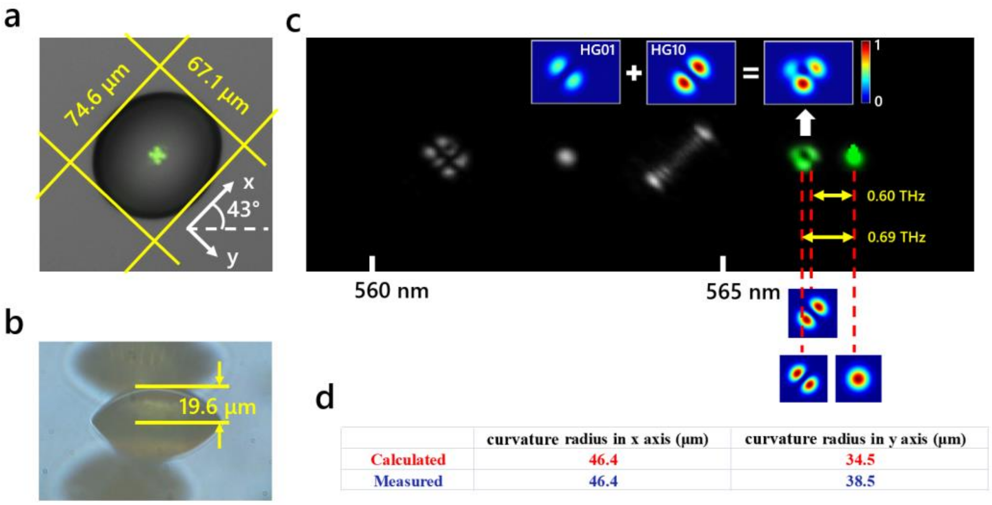

	curvature radius in x axis (μm)	curvature radius in y axis (μm)
Calculated	46.4	34.5
Measured	46.4	38.5

Fig. S2. Laser modes from an elliptical droplet. (a) Top-view of an elliptical droplet. *x* and *y* axes denote the long and short axes of the droplet. **(b)** Side-view of the elliptical droplet in (a). **(c)** Hyperspectral image of laser modes from the elliptical droplet. Insert: simulation pattern that is composed of HG_{01} and HG_{10} modes with orthogonal long axes. **(d)** Calculated curvature radii along *x* and *y* directions of the elliptical droplet based on laser modes (red) and measured curvature radii along *x* and *y* directions of the elliptical droplet (blue).

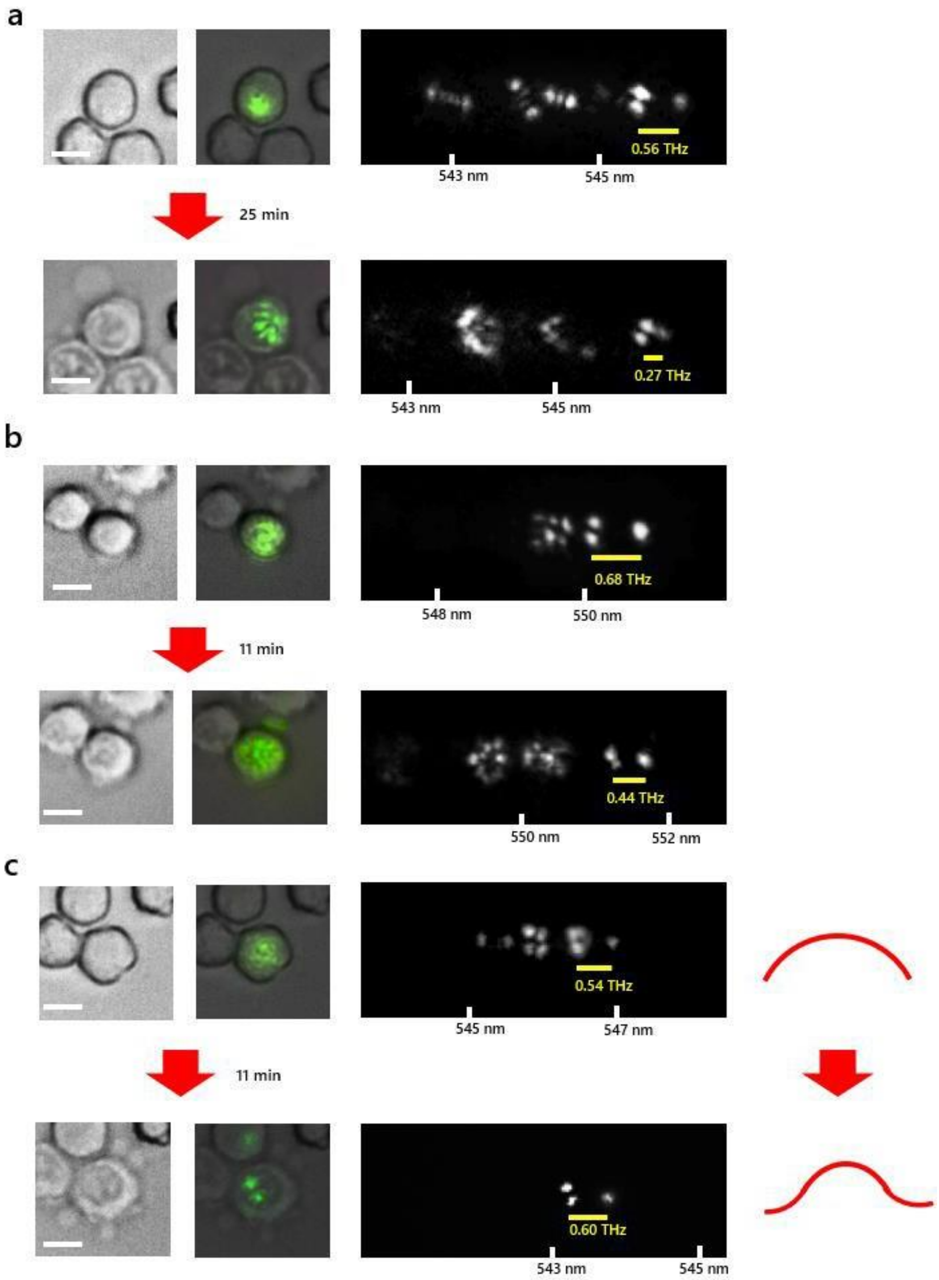

Fig. S3. Evolutions of laser modes from three individual live C2C12 cells with time. Left panel, bright-field images of cells; middle panel, far-field laser emission patterns superimposed on the bright-field images; right panel, hyperspectral images of laser modes. In **(a)** and **(b)**, the frequency spacings between TEM_{01} and TEM_{00} modes decreased with time. In **(c)**, the frequency spacing between TEM_{01} and TEM_{00} modes increased slightly in 11 minutes, indicating the increase of local curvature of the cell. At the right-side of (c), the red curves illustrated the possible profile changes of the cell with spreading, showing increasing local curvature. Scale bar: 10 μm.

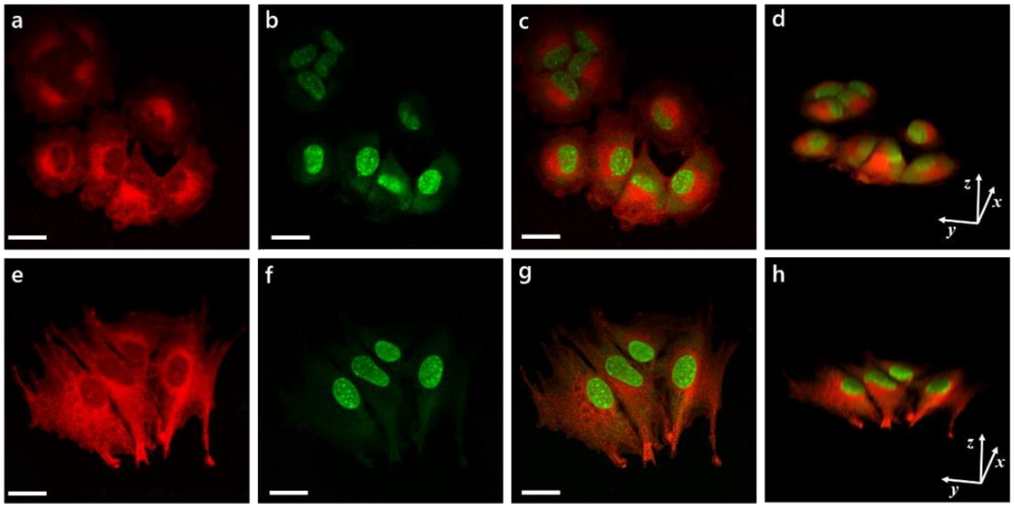

Fig. S4. Confocal microscopic images of C2C12 cells by staining membranes and nucleus. (a-d) Confocal microscopic images of C2C12 cells with spreading time of 2 hours. **(a)** Confocal microscopic image of the cells by staining membranes. **(b)** Confocal microscopic image of the cells by staining nucleus. **(c)** Emerged image of (a) and (b). **(d)** 3D confocal microscopic image. **(e-h)** Confocal microscopic images of C2C12 cells with spreading time of 6 hours. **(e)** Confocal microscopic image of the cells by staining membranes. **(f)** Confocal microscopic image of the cells by staining nucleus. **(g)** Emerged image of (e) and (f). **(h)** 3D confocal microscopic image. DIO and YOPRO dyes were utilized to stain cell membranes and nuclei, respectively. Scale bar: 20 μm

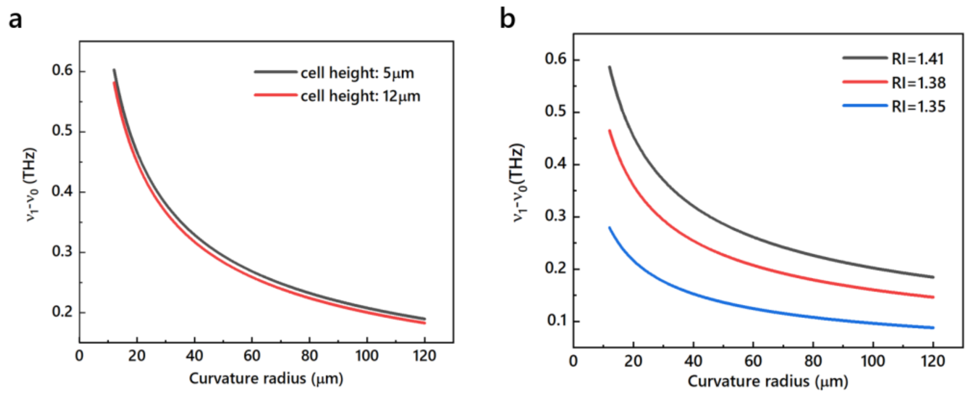

Fig. S5. (a) Calculated frequency spacing between TEM_{01} and TEM_{00} modes as a function of local curvature radius under the cell height of 5 μm and 12 μm. The two curves are close to each other, indicating that the frequency spacing is not sensitive to cell height. Refractive index (RI) of cells: 1.41; RI of surrounding environment: 1.334; cavity length: 16 μm. **(b)** Calculated frequency spacing between TEM_{01} and TEM_{00} modes as a function of local curvature radius under the cell RI of 1.35, 1.38 and 1.41. Cell height: 10 μm; cavity length: 16 μm.

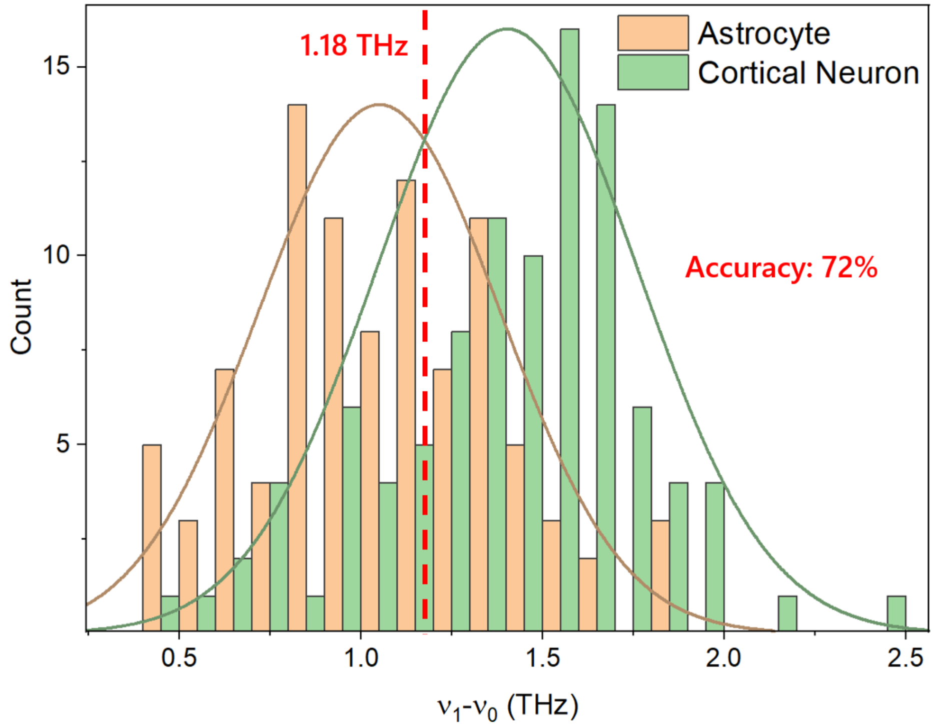

Fig. S6. Statistics of frequency spacings between TEM_{01} and TEM_{00} modes from cortical neurons and astrocytes. By choosing 1.18 THz as the classification criterion, the classification accuracy was 72 %. Sample size: n=99 and 95 for neurons and astrocytes, respectively.

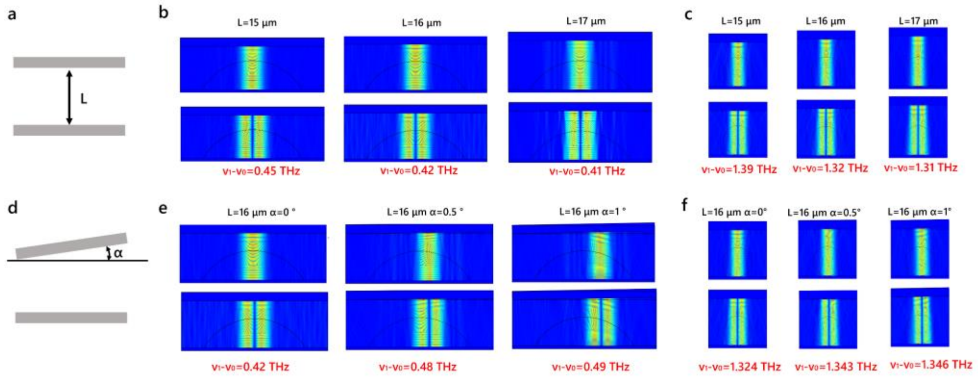

Fig. S7. Simulations of transverse-mode frequency spacings under variations of cavity lengths (a-c) and tilting angles of top mirrors (d-f). (a) Schematic of FP cavity with varying cavity length (L). **(b)** Electric field distributions of TEM_{00} and TEM_{01} modes and frequency spacings (v_1-v_0) under the cavity lengths of 15 μm, 16 μm, and 17 μm based on adherent cell models (hemispheres). **(c)** Electric field distributions of TEM_{00} and TEM_{01} modes and frequency spacings (v_1-v_0) under the cavity lengths of 15 μm, 16 μm, and 17 μm based on suspension cell models (spheres). **(d)** Schematic of FP cavity with varying tilting angle of a mirror (α). **(e)** Electric field distributions of TEM_{00} and TEM_{01} modes and frequency spacings (v_1-v_0) under the tilting angles of 0°, 0.5°, and 1° based on adherent cell models. **(f)** Electric field distributions of TEM_{00} and TEM_{01} modes and frequency spacings (v_1-v_0) under the tilting angles of 0°, 0.5°, and 1° based on suspension cell models. In (b) and (e), the curvature radii and heights of the hemispheres are 20 μm and 10 μm, respectively. In (c) and (f), the radii of the spheres are 5 μm. RI of hemispheres and spheres is 1.41. RI of surrounding environments is 1.334.

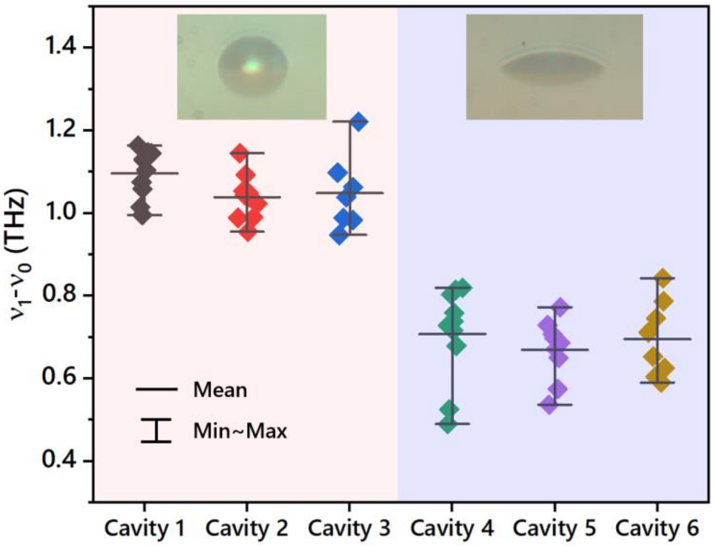

Fig. S8. Transverse-mode frequency spacings under different cavities. Cavity 1, 2, and 3 were formed by building FP cavities three times repeatedly based on a fixed bottom mirror with a hydrophobic surface. Droplets with diameters ranging from 34 μm~38 μm were selected for the statistics of transverse-mode frequency spacings. Cavity 4, 5, and 6 were formed by building FP cavities three times repeatedly based on another fixed bottom mirror with a hydrophilic surface. Droplets with diameters ranging from 49 μm~55 μm were selected for the statistics of transverse-mode frequency spacings. Microbeads with 27 μm-diameters were used as spacers. Insets: microscopic images of droplets in side-view.

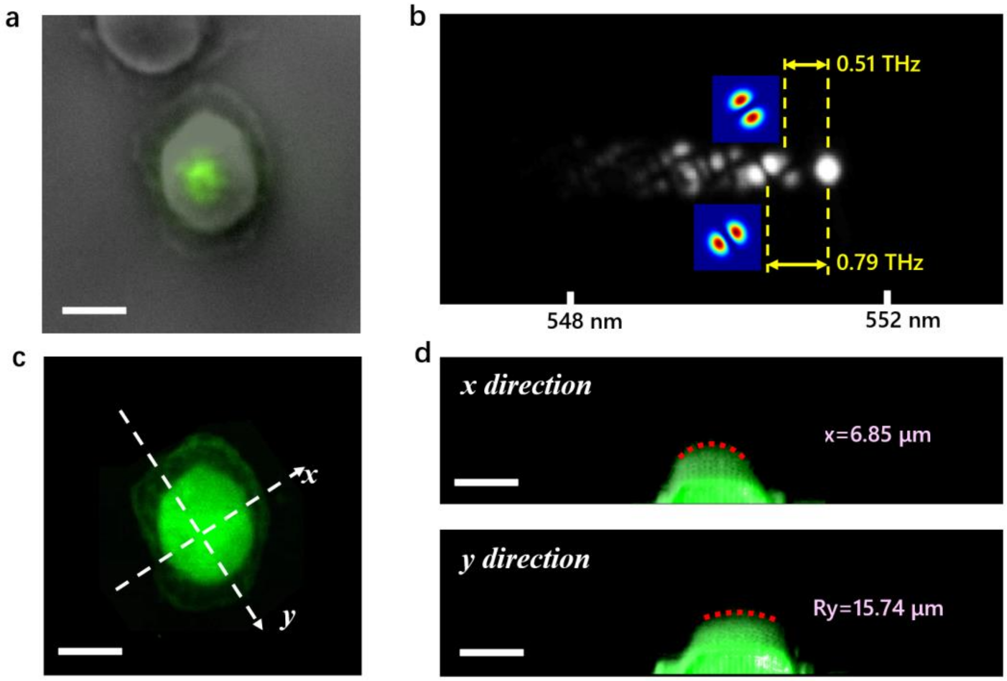

Fig. S9. Laser modes from a fixed C2C12 cell. (a) Far-field laser emission pattern superimposed on the bright-field image of a cell. **(b)** Hyperspectral image of laser modes. Insert: simulation patterns of HG_{01} and HG_{10} modes with orthogonal long axes. **(c)** Confocal microscopy image of the cell. *x* and *y* directions denote the long-axis directions of HG_{01} and HG_{10} modes in (b). **(d)** Cell profiles from the cross-section planes along *x* and *y* directions in (c). Red curves illustrated the local profiles with the curvature radii calculated based on the frequency spacings of laser modes in (b). Scale bar: 10 µm.

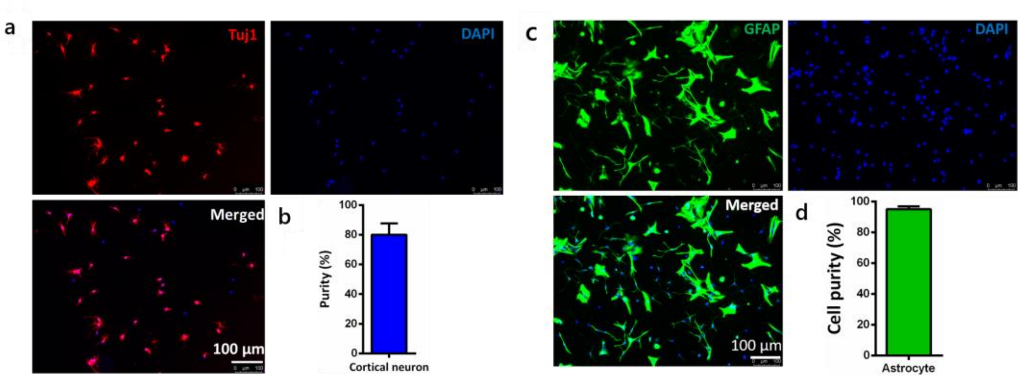

Fig. S10. The purity of cortical neurons (a-b) and astrocytes (c-d). (a) Representative fluorescent images of Tuj1 stained cells. (b) The purity of cortical neurons. It was calculated by using the number of Tuj1+ cells divided by the total number of cells x 100%. Three biological repeats were included. (c) Representative fluorescent images of GFAP stained cells. (d) The purity of astrocytes. It was calculated by using the number of GFAP+ cells divided by the total number of cells x 100%. Three biological repeats were included. Data presentation: mean ±SD.
